# Oscillatory, Computational, and Behavioral Evidence for Impaired GABAergic Inhibition in Schizophrenia

**DOI:** 10.1093/schbul/sbz066

**Published:** 2019-06-20

**Authors:** Alexander D Shaw, Laura Knight, Tom C A Freeman, Gemma M Williams, Rosalyn J Moran, Karl J Friston, James T R Walters, Krish D Singh

**Affiliations:** 1 CUBRIC, School of Psychology, College of Biomedical and Life Sciences, Cardiff University, Cardiff, UK; 2 MRC Centre for Neuropsychiatric Genetics and Genomics, Division of Psychological Medicine and Clinical Neurosciences, School of Medicine, Cardiff University, Cardiff, UK

**Keywords:** schizophrenia, GABA, inhibition, dysconnection, oscillatory, behavioral

## Abstract

The dysconnection hypothesis of schizophrenia (SZ) proposes that psychosis is best understood in terms of aberrant connectivity. Specifically, it suggests that dysconnectivity arises through aberrant synaptic modulation associated with deficits in GABAergic inhibition, excitation–inhibition balance and disturbances of high-frequency oscillations. Using a computational model combined with a graded-difficulty visual orientation discrimination paradigm, we demonstrate that, in SZ, perceptual performance is determined by the balance of excitation–inhibition in superficial cortical layers. Twenty-eight individuals with a DSM-IV diagnosis of SZ, and 30 age- and gender-matched healthy controls participated in a psychophysics orientation discrimination task, a visual grating magnetoencephalography (MEG) recording, and a magnetic resonance spectroscopy (MRS) scan for GABA. Using a neurophysiologically informed model, we quantified group differences in GABA, gamma measures, and the predictive validity of model parameters for orientation discrimination in the SZ group. MEG visual gamma frequency was reduced in SZ, with lower peak frequency associated with more severe negative symptoms. Orientation discrimination performance was impaired in SZ. Dynamic causal modeling of the MEG data showed that local synaptic connections were reduced in SZ and local inhibition correlated negatively with the severity of negative symptoms. The effective connectivity between inhibitory interneurons and superficial pyramidal cells predicted orientation discrimination performance within the SZ group; consistent with graded, behaviorally relevant, disease-related changes in local GABAergic connections. Occipital GABA levels were significantly reduced in SZ but did not predict behavioral performance or oscillatory measures. These findings endorse the importance, and behavioral relevance, of GABAergic synaptic disconnection in schizophrenia that underwrites excitation–inhibition balance.

## Introduction

The neurobiological underpinnings of schizophrenia (SZ) are currently poorly understood, with several competing synaptic hypotheses; including dysfunction of GABAergic neuromodulation,^1^ NMDA receptor hypofunction (review^2^) and aberrant dopamine regulation.^3^ Reconciling these various models, the dysconnection hypothesis^4^ proposes that SZ results from synaptic dysconnectivity—both between brain regions and between the layers of cortical columns—caused by aberrant synaptic modulation. Physiologically, synaptic modulation refers to the balance of excitation and inhibition, whereby GABAergic neurons exert control (and synchrony) over the firing of principal cells in cortex. In rodent, NMDA receptor hypofunction reduces excitation of GABAergic cells, which results in a disinhibited, hyperdopaminergic state.^5^ As such, cortical deficits in NMDA, GABA, or both, may underlie the pathophysiology of schizophrenia.

GABAergic theories of SZ have recently gained support from genetic studies, including evidence for a primary GABAergic hypothesis, rather than secondary, by implication in large scale studies of both rare^6^ and common^7^ genetic variation. In vivo studies of altered bulk GABA concentrations in schizophrenia are, however, equivocal, with a recent meta-analysis suggesting no detectable differences in GABA levels across prefrontal, parietal/occipital cortex, and striatum using proton magnetic resonance spectroscopy (^1^H-MRS).^8^

Performance on visual orientation discrimination paradigms is known to correlate with occipital GABA concentrations, making these paradigms useful probes of the GABAergic system^9^ without putative confounding effects from dopaminergic influences. Performance tends to correlate with peak oscillation frequency in the gamma range (30+ Hz) in the visual cortex.^9^ Because gamma oscillations are also dependent on excitation–inhibition balance—under the pyramidal-interneuron gamma (PING) model of oscillatory activity^10,11^—gamma oscillations have been proposed as a consequence of the synaptic modulatory mechanisms thought to be disrupted in SZ.^12^

Neurophysiologically, the PING model proposes that gamma frequency oscillations are generated in superficial layers (2/3) of cortex and mediated by fast GABA-A and AMPA receptors as well as slower NMDA receptors.^13^ Deficits in GABA may therefore explain reports of reduced gamma oscillatory power in SZ.^11,14–16^

Here, we address the relationship between gamma oscillations and orientation discrimination performance in SZ, using an integrated framework. Using neurophysiologically informed modeling based on dynamic causal modeling (DCM), we demonstrate how GABA-mediated local dysconnectivity leads to altered gamma oscillatory activity that predicts behavioral performance in a graded, disease-specific manner.

## Materials and Methods

### Participant Sample and Recruitment

Participants consisted of 28 schizophrenia participants (8 female, mean age males = 44.2 [SD = 8.6] years, females = 45.9 [SD = 8] years) and 30 control participants (11 female, mean age males = 42.4 [SD = 10] years, females = 40.6 [SD = 10.2] years). The age range was 22–58 for the SZ group and 24–58 for the control group. There was no difference in age between the groups (*P* > .1). Participants were recruited from the Cognition in Psychosis study (Schizophrenia Working Group of the Psychiatric Genomics Consortium^17^), with full details of the case sample recruitment and procedures are provided in Lynham et al.^18^ Controls were recruited primarily through an advert placed on the Cardiff University Noticeboard system and opportunistically from CUBRIC, Cardiff University. This study was approved by the NHS ethics Board and also the Cardiff University School of Psychology Ethics Board. Full details of the inclusion and exclusion criteria can be found in the supplementary materials.

### Successful Data Collection

One male control participant was unable to undertake either the MEG experiment reported here or a GABA MR Spectroscopy assessment, but did complete the behavioral orientation discrimination experiment. Similarly, 2 SZ participants (1 male, 1 female) did not complete the MEG data collection, but MRS data and behavioral data were successfully acquired. This means for the multimodal comparisons reported here, the total cohort sizes were 29 controls and 27 SZ participants. The demographics of these participants are outlined in table 1. Details of the acquisition parameters of the GABA MRS scan are found in supplementary materials.

**Table 1.** Sample Ages by Group and Gender

Group	Number	Mean Age (y)	SD Age (y)	Independent *T*-Test SZ Vs Controls (*P* value)
All controls	29	41.1	10.1	
All SZ	27	44.7	8.5	0.16
Female controls	11	40.2	12.1	
Female SZ	8	45.9	8.0	0.3
Male controls	18	41.6	9.4	
Male SZ	19	44.2	8.8	0.4

### Behavioral Visual Orientation Discrimination Task

**Fig. 1.**
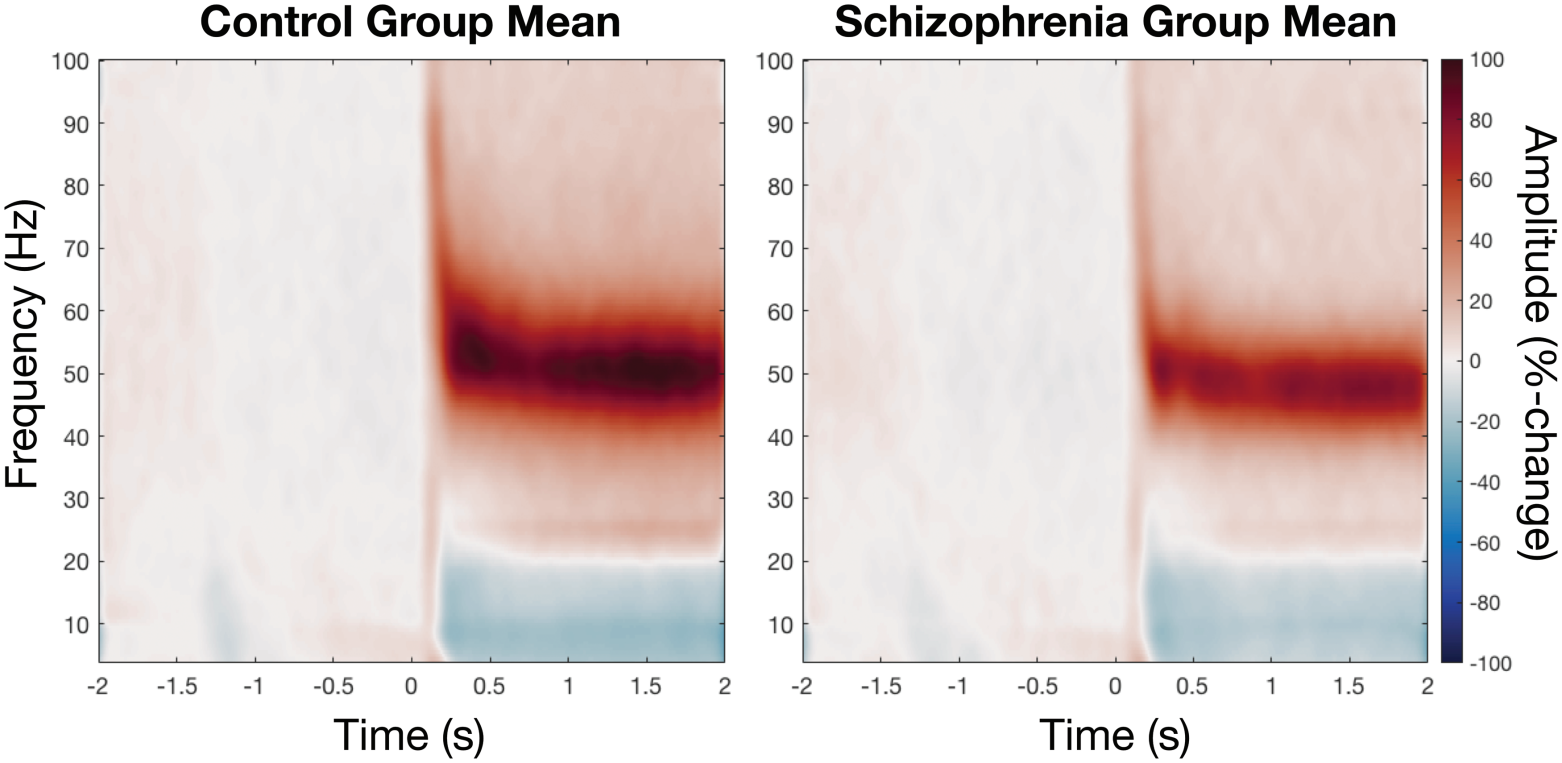
Group averaged, percentage-change time–frequency spectrograms, with visual grating pattern onset at 0 s. Both the transient and sustained gamma responses are evident, with marginal reductions in the SZ group, compared with the control group.

Orientation discrimination thresholds were measured using a 2-alternative forced choice procedure as shown in figure 1A. Stimuli were programmed using DELPHI and consisted of 2 sequentially presented circular grating patches (width 4°, contrast 80%, 3 cycles per degree, refresh rate 80 Hz, mean luminance 44.5 cd/m^2^). The task used 2 stimulus types, an easier vertical condition in which the orientation difference between the 2 patches varied around a constant mean of 0° and a more difficult oblique condition where the orientation between the 2 patches varied around a constant mean of 45°. Further details on this paradigm can be found in the supplementary materials.

### Gamma Acquisition and Analysis

The MEG paradigm was based on the paradigm of Hoogenboom,^19^ where stimuli consisted of a centrally presented, circular sine wave grating (diameter 5°, spatial frequency 2 cpd and maximum contrast). The circular grating drifted inwards toward the fixation point and the speed of this contraction increased (velocity step at 2.2 deg/s) at a randomized time of between 50 and 3000 ms after stimulus onset. The participant was instructed to press a button with their right index finger as soon as they noticed a speed change. Stimulus offset was followed by a resting period of 1000 ms, in which subjects were given visual feedback of either “OK,” “early,” or “late” depending on their response. For further details on this paradigm, see supplementary materials.

MEG data were collected using a 275-channel CTF MEG system sampled at 1200 Hz. Datasets were epoched into trials of 4 s centered on stimulus onset. Trials were visually inspected for gross recording artifact on a trial-by-trial basis. Synthetic aperture magnetometry (SAM) beamformer^20^ analysis was used to compare activity for 2 s of baseline (−2 to 0 s) and 2 s of visual stimulation (0 to 2 s). Pseudo *t*-statistics were used to compute differences between baseline and stimulation activity. Volumetric SAM images were computed in a 30–80 Hz frequency band. The peak location of gamma activity in each participant’s visual cortex, defined by the peak occipital *t*-statistic, was located and virtual sensors constructed for these locations using covariance matrices low-pass filtered at 100 Hz. Time-frequency analysis of these virtual sensors used Hilbert envelopes of bandpass filtered data^21^ between 1 and 100 Hz in 0.5 Hz steps to produce spectra in which the responses at each time/frequency point were expressed as mean percentage change from baseline. The peak frequency and amplitude spectral features used for statistical analysis were derived by identifying the peak after averaging the Hilbert envelope at the time window of interest (0–300 ms for transient responses, 300–800 ms for sustained/induced responses). Accordingly, peak frequency measures are in units of hertz (Hz), while amplitude measures are in units of percentage-change from baseline (%).

### Neurophysiologically Informed Modeling

Using a convolution-based canonical microcircuit model (CMC),^22^ optimized to reflect the known properties of primary visual cortex (V1) and implemented within the dynamic causal modeling (DCM) framework of SPM8 (https://www.fil.ion.ucl.ac.uk/spm/software/spm8/), we modeled the spectral density recorded by the MEG virtual sensors, to better characterize the neuronal population-level interactions underlying the oscillatory responses. A brief overview of the modeling framework is included in the supplementary materials.

Within the CMC, responses were driven by endogenous afferent input to spiny stellate cells, formed of a mixture of scale free fluctuations. The CMC featured 4 interconnected cell populations (layer 2/3 and 5/6 pyramidal cells, layer 4 spiny-stellate cells and multi-layer inhibitory interneurons; figure 5A), each modeled by parameterized first-order differential equations describing the evolution of states (membrane potentials and postsynaptic currents). Model parameters control the strength of connections between the cell populations, the synaptic time constants of these interactions and parameters encoding the form of endogenous input. This model has previously been used to recover perturbations of neuronal population-level dynamics induced by GABAergic manipulation following administration of tiagabine,^22^ making it a sensitive and specific model for assaying cortical GABAergic dynamics. Coupled with a weighted-contribution observation model,^22^ the model was inverted to fit the spectral density of the virtual sensor over the 4–90 Hz range using DCM for cross spectral responses.^23^

### Statistical Analyses

Details of the statistical analyses performed are described in supplementary materials. All results are Bonferroni corrected for multiple comparisons.

## Results

### Gamma Frequency and Amplitude Are Reduced in Schizophrenia

**Fig. 2.**
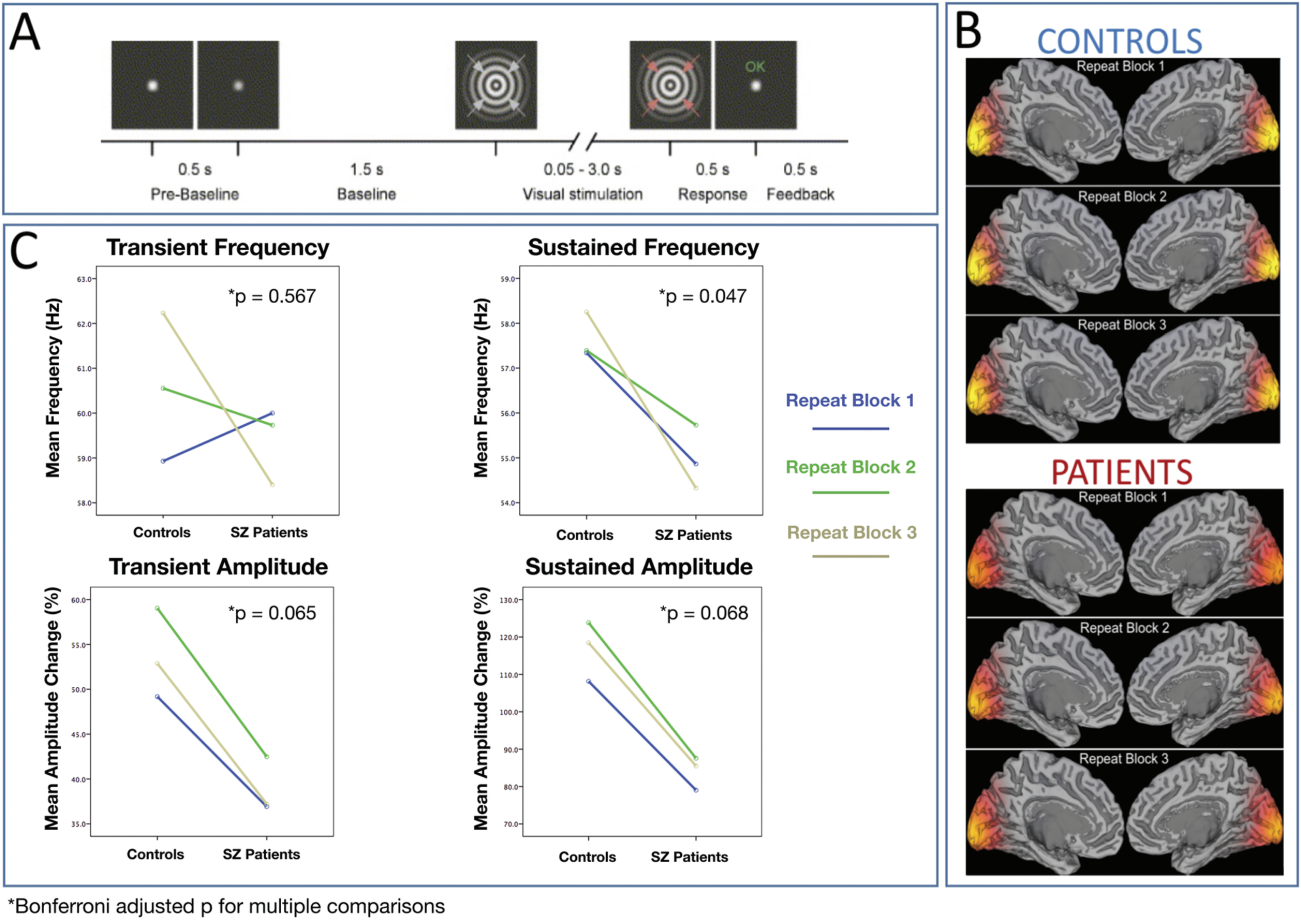
(A) Schematic of the stimuli and trial timings. (B) Group SAM beamformer reconstructions of the stimulus-induced gamma changes (30–80 Hz) during the sustained stimulus presentation time (50–3000 ms). For both participant cohorts, these group SAM maps are shown on an MNI template brain, for each of the three experimental runs. Bilateral gamma increases were seen in or close to the calcarine sulcus. (C) Quantification of SZ-related differences in gamma amplitude and frequency. These were assessed separately for the transient broadband gamma “spike” (0–300 ms) and the initial sustained gamma period (300–800 ms).

Group SAM beamformer reconstructions—with weights optimized for the gamma range—demonstrated bilateral gamma increases in or close to the calcarine sulcus, consistent with a primary visual cortex generator (figure 2B).

**Fig. 3.**
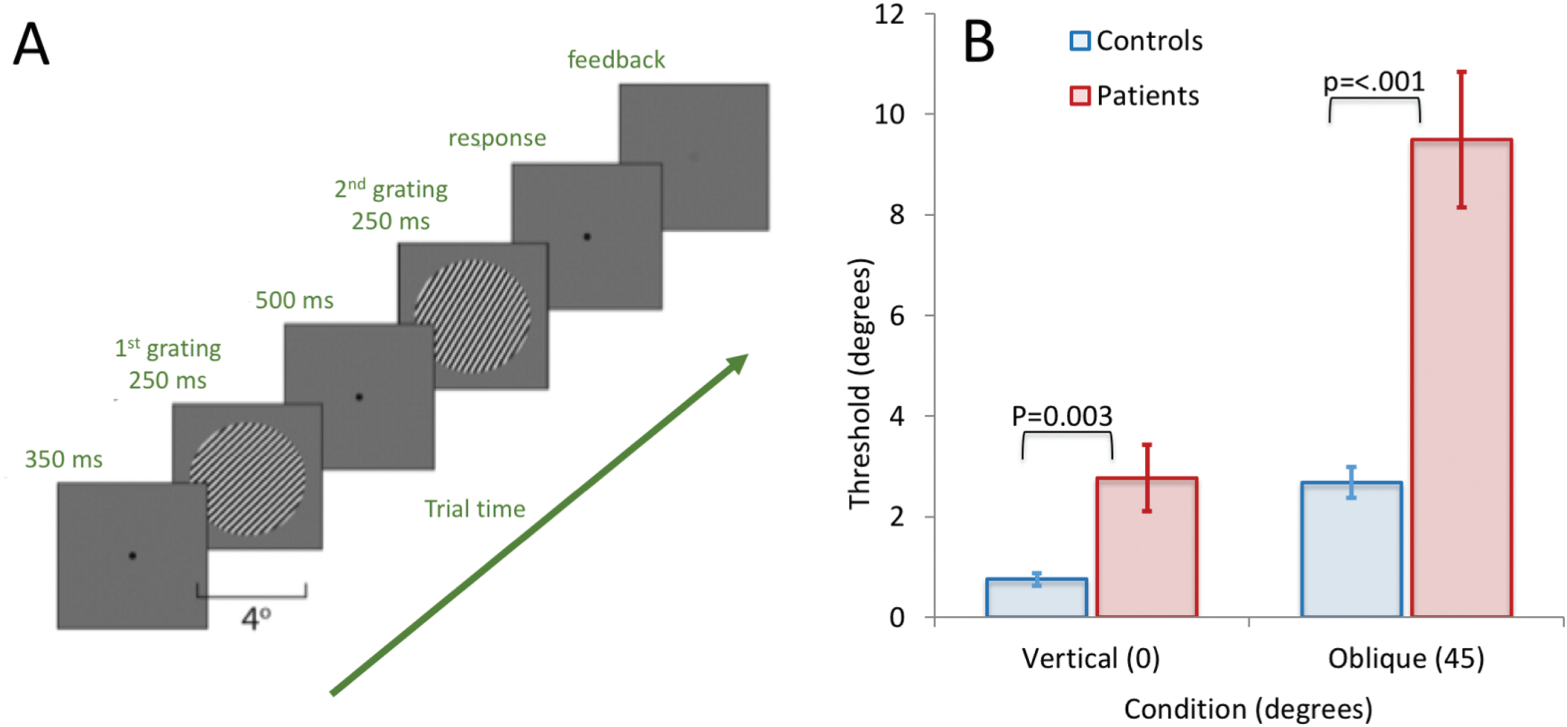
(A) Participants viewed a sequential presentation of 2 circular grating patches, each presented for 250 ms at a frame rate of 80 Hz, with a grating contrast of 80%, a spatial frequency of 3 cycles/degree, a mean luminance of 44.5 cd/m^2^ and subtending 4° of visual angle. Participants were instructed to respond which of the 2 sequentially presented gratings was oriented more to the right in a 2-alternative forced choice design. (B) Results of the task for SZ and controls in both vertical and oblique conditions.

Two-way repeated measures ANOVAs were used to determine between-group differences in peak oscillatory features, calculated as percentage-change. A main effect of group was observed for sustained (induced) frequency [*F*(1, 29) = 4.15, *P* = .047] with the SZ group showing an estimated mean reduction of 3 Hz (Controls mean = 58, SE = 0.92, SZ mean = 55, SE = 0.95). Trends in the same direction were observed for both transient amplitude [*F*(1, 29) = 3.6, *P* = .065] and sustained amplitude [*F*(1, 29) = 3.5, *P* = .068]. This is most evident in the group-mean time-frequency representations of figure 3. No differences in prestimulus gamma features were observed between groups (supplementary materials).

### Orientation Discrimination Performance Is Impaired in Schizophrenia

A 2-by-2 ANOVA was used to examine between group differences in orientation discrimination performance. This revealed a group-by-condition interaction effect [*F*(1, 28) = 8, *P* = .007]. A significant effect of condition (vertical vs oblique) demonstrated “*the oblique-effect*,” in which performance is much poorer for oblique targets [*F*(1, 28) = 16.34, *P* < .001]. A significant effect of group revealed that the SZ group were significantly impaired (higher thresholds) across conditions [*F*(1, 28) = 20.9, *P* < .001].

### Impairments of Superficial Pyramidal-Interneuron Connectivity Predict Negative Symptom Severity

Group differences in synaptic connectivity parameters were assessed using repeated-measures ANOVA (left columns of table 2). There was no effect of session (radial 1–3) [group-by-session-by-parameter *F*(4.429) = 0.54, *P* = .73, parameter-by-session *F*(4.429) = 2.18, *P* = .06) but a parameter-by-group effect (*F* = 4.893, *P* < .001).

**Table 2.** Group Effects on Parameters

Parameter	*F* (ANOVA)	*P* (ANOVA)	BF (*T*-Test)	BF (Best)/BF
G4	8.583	0.005*	8.3	1.0
G7	5.683	0.021	2.7	3.0
G6	5.0773	0.029	2.1	3.9
G12	4.039	0.05	1.4	5.8
G8	0.686	0.411	0.4	22.7
G9	0.561	0.457	0.3	23.9
G11	0.449	0.506	0.3	25.0
G5	0.048	0.828	0.3	29.5

*P = survives Bonferroni correction.

Main effects of group on parameter were observed for G4 (*P* = .005), G6 (*P* = .029), G7 (*P* = .021) and G12 (*P* = .05), table 2, indicating reductions of connectivity strength in the SZ group. Only G4 was significant after Bonferroni correction for the number of coupling parameters (8 parameters, G4 *P* = .04).

We used JASP to compute Bayes factors (BF) for each parameter (right side of table 2, unpaired *t*-test, averaging over sessions as no session effect). This revealed that the G4 parameter had the highest BF (8.3, “moderate”), followed by G7 (BF = 2.7, “weak”). The relative evidence for G4 having a stronger effect than G7 (and hence any other parameter) had a Bayes Factor of 3.0, ie, “moderate.”

**Fig. 4.**
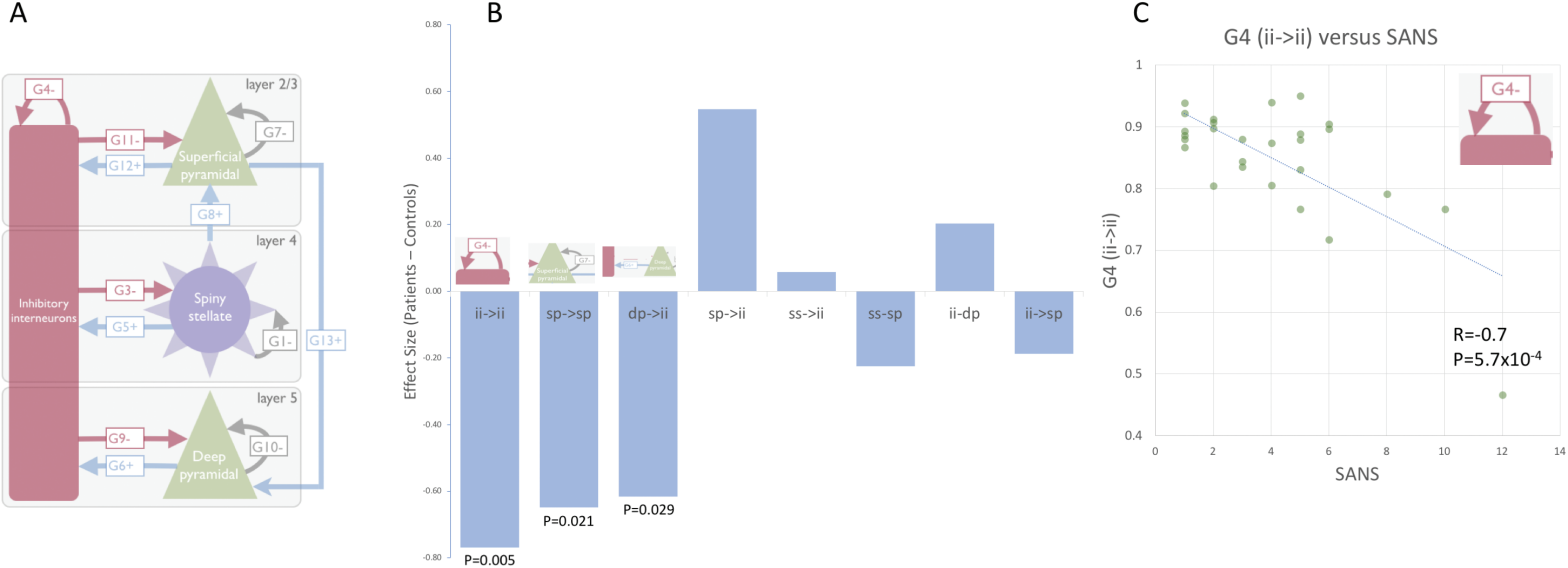
(A) DCM of the MEG data was performed using a CMC comprising 4 neural populations, with plausible neurophysiological connections and intrinsic time-constants. (B) Demonstrating the difference in synaptic connection strengths between SZ and controls. (C) The strength of inhibitory self-modulation of inhibitory interneuron populations predicts negative symptom score (SANS) in SZ (*r* = −.7, *P* = 5.7e-4, Bonferroni corrected *P* = .0046).

The strength of the G4 parameter showed a significant negative correlation with negative symptom score, as measured with the SANS (*r* = −.7, *P* = 5.7e-04, Bonferroni corrected 0.0046) (figure 4C).

### Behaviorally Relevant, Disease-Related Dysconnectivity of Local GABA Function

**Fig. 5.**
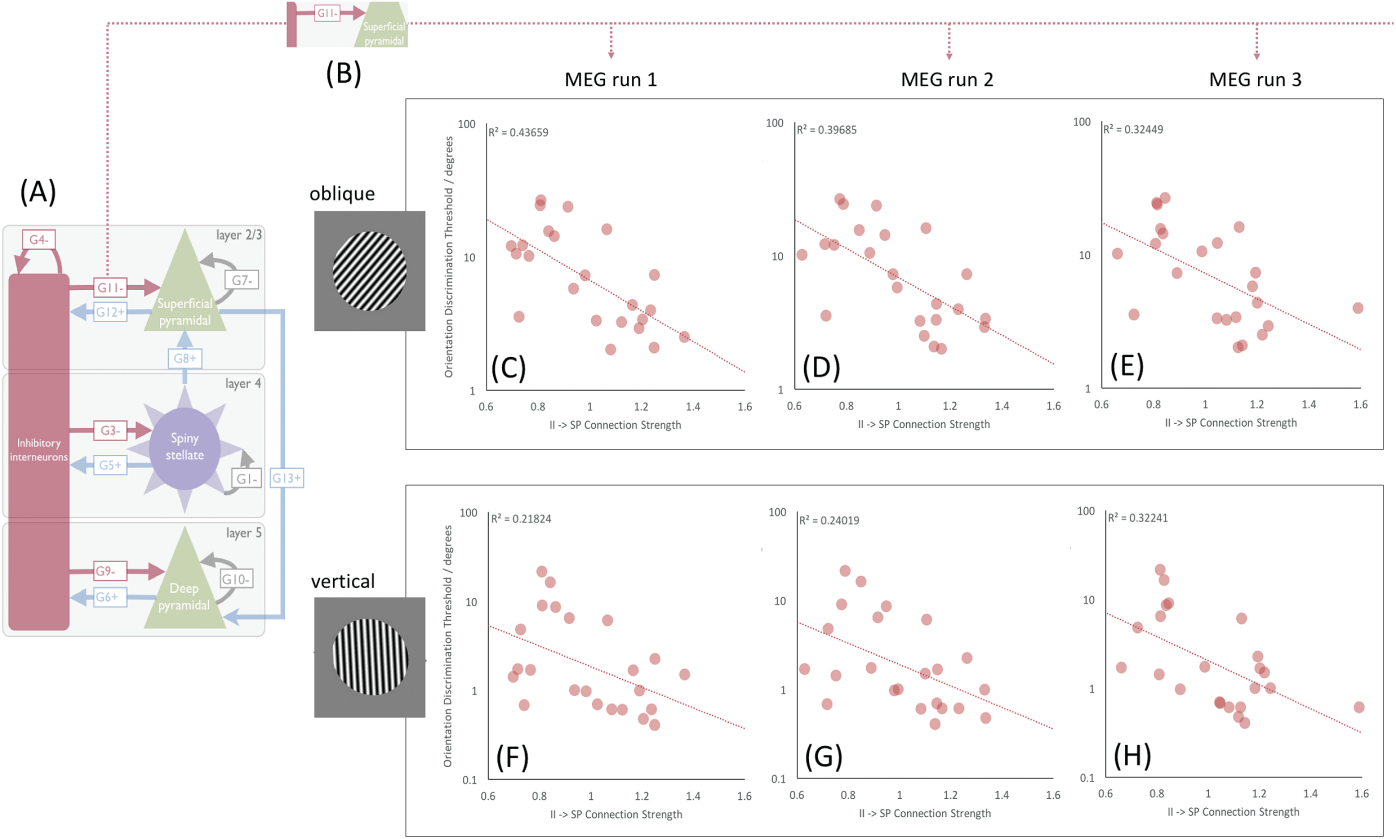
Demonstrating the negative correlation between synaptic coupling strength of inhibitory interneurons and superficial pyramidal cells (G11) and performance on each of the trials, for both vertical and oblique conditions, for the SZ group.

The inhibitory connection between the inhibitory interneurons and the superficial pyramidal cell population (G11, shown in figure 5B) consistently predicted behavioral performance, in the SZ group, across the 3 MEG sessions. Bayes factors for each parameter demonstrated strong (oblique session 1 and 2), moderate (oblique session 3) and anecdotal evidence (vertical sessions 1–3) for G11, but no evidence for any other parameters (table 3), with anecdotal evidence in some for the null. Note that the relative evidence for G11 over all other parameters is strong in all oblique sessions (BFG11/BF >12).

**Table 3.** Bayes Factors for Predicting Performance in the SZ Group

Parameter		G4	G5	G6	G7	G8	G9	G11	G12
Oblique	Session 1	0.264	0.329	0.259	0.288	0.31	0.392	11.354^a^	0.297
	Session 2	0.366	0.267	0.319	0.281	0.302	0.696	12.029^a^	0.262
	Session 3	0.521	0.28	0.32	0.415	0.495	0.702	8.482^b^	0.275
Vertical	Session 1	0.259	0.364	0.314	0.28	0.289	0.321	1.239	0.285
	Session 2	0.288	0.3	0.259	0.329	0.275	0.421	1.499	0.372
	Session 3	0.343	0.259	0.89	0.498	0.349	0.967	2.961	0.77

^a^BF > 10 = “strong” evidence.

^b^BF3-10 = “moderate.”

In figures 5C–E, the correlation between G11 parameter and behavioral performance is shown for the oblique version of the task. Cases with stronger inhibitory connections (II → SP) were better at the task, with this model parameter explaining 44%, 40%, and 32% of the variance in the behavioral performance when derived from MEG runs 1, 2, and 3, respectively. By using the 3 runs separately, we demonstrate repeatability of this result within the sample. These model results demonstrate the key importance of local, GABA-mediated, connectivity in determining low-level visual performance in this SZ cohort.

## Discussion

Using MEG imaging and DCM to “computationally assay” cortical GABAergic inhibition in the visual cortex in SZ, we have demonstrated that synaptic disinhibition of superficial layer pyramidal populations (in silico) predicts performance during an orientation discrimination task, in a graded and disease-specific manner.

Under the disconnection hypothesis of SZ, hypofunction of NMDA receptors leads to reduced activation of GABAergic interneurons, which in turn results in disinhibition of pyramidal cells.^5,24^ This aberrant state means impaired synchrony, which in turn suggests impaired oscillatory activity. We demonstrate significant reductions in the peak frequency of induced gamma oscillations in SZ (corrected *P* = .047), along with trends toward reduced amplitude (corrected *P* = .065), in accordance with previous reports.^25^ Moreover, as discussed in the supplementary materials, we demonstrated a reduction in the total concentration of GABA in the same region; however, this did not correlate with reductions of gamma frequency (*r* = −.1) or amplitude (*r* = −.13).

Employing a neurobiologically grounded neuronal model to examine population interactions underlying visual cortical responses, we find reductions in key synaptic parameters in SZ (in silico), which include inhibitory modulation of inhibitory interneuron and superficial pyramidal populations as well as excitatory projections from deep pyramidal to interneuron populations, although only the former survive correction for multiple comparisons. Notably, the synaptic parameters related to granular and deep cells (layers 4, 5/6) did not show this association with gamma.

Assessing the relationship between synaptic parameters and performance during orientation discrimination, we find that the synaptic strength of inhibitory connections from inhibitory interneurons to superficial layer pyramidal cells (II → SP, G11, figure 5) predicts performance across 3 runs of the task. Strikingly, this parameter corresponds to the aforementioned mechanism of disinhibition and has been reported as a key determinant of gamma frequency.^22,26^ As such, our findings formalize a framework whereby disturbances of gamma, and impairments in orientation discrimination performance may be understood as consequences of GABAergically mediated dysconnectivity in SZ.

### Gamma and Task Performance Deficits

Peak gamma frequency was reduced in SZ for sustained-induced (nonstimulus locked) responses, and a trend was observed in the same direction for amplitude, however this did not survive Bonferroni correction (corrected *P* = .065). Both the frequency and amplitude of gamma oscillations in visual cortex are thought to reflect the encoding of stimulus properties^27^ and feature integration,^28^ while peak frequency is known to depend on both the size of primary visual cortex (V1)^29^ and the density of GABA receptors in V1.^30^ As such, gamma frequency is interesting in the context of perceptual diseases such as SZ (reviewed in ref.^31^), because it represents an intermediate phenotype linking cortical physiology and cognition.

Reduced peak gamma frequency in SZ has been reported during perception of Gestalt visual stimuli, using EEG,^32^ leading to the proposition that reduced oscillatory frequency is a specific index of impaired feature-binding processes.^32^ Our results extend these neural correlates of perception; demonstrating reductions in primary visual cortex gamma frequency in SZ are also induced by inward-drifting annular grating stimuli.

The amplitude of the initial evoked gamma “spike” demonstrated a trend to toward reduction in the SZ group, however this did not survive correction for multiple comparisons (corrected *P* = .065). Given reduced amplitude reflects reduced synchrony, this trend aligns with the aforementioned NMDAR mediated disinhibition hypothesis, whereby NMDAR hypofunction leads to disinhibition of pyramidal cells^5^ by GABAergic interneurons, which leads to aberrant activity, and therefore reduced synchrony. This finding is also in accordance with previous reports of reduced gamma amplitude in SZ during face processing,^33^ suggesting this result is generalizable to all visual processing and not stimulus-specific.

It is plausible that the SZ group differed from the controls in their attentiveness to the MEG visual paradigm, despite the button press required on each trial. However, attention has been shown not to modulate sustained low-frequency gamma oscillatory responses in *primary* (V1) visual cortex.^34^

### Modeling

Neurophysiologically informed modeling suggested local dysconnectivity in SZ, mediated by reduced inhibitory gain control of inhibitory interneuron populations (G4, corrected *P* = .04, BF = 8.3), and trends toward reduced inhibitory gain control of superficial pyramidal cells (G7, *P* = .021, corrected *P* = ns) and the excitatory projection from deep layer pyramidal cells to inhibitory interneurons (G6, *P* = .029, corrected *P* = ns). Both G4 and G7 are inhibitory connections located in superficial cortical layers and are major determinants of gamma amplitude and frequency, respectively.^22^

Interactions of these populations underlie the “disinhibition hypothesis,”^5,35^ whereby hypofunction of NMDA receptors on interneurons results in pyramidal cells reaching a disinhibited, aberrant state. Thus, within the context of our model, we find evidence linking the disinhibition hypothesis of SZ with altered gamma oscillations. An analysis of parameter Bayes factors, to assess each parameter in explaining group, revealed that the G4-only model had the most evidence (BF = 8.3), with moderate evidence that this was better than the next best parameter, G7 (BFG4/BFG7 = 3.1).

In the SZ group, the GABAergic synaptic connection from interneurons to superficial pyramidal cells (G11) was found to predict orientation discrimination performance across 3 repeated runs, although parameter Bayes factors indicated strong and moderate evidence only for the oblique (hard) condition. This is important, because this synaptic parameter is instrumental in the generation of gamma oscillations,^10,26^ mediated by GABAergic inhibition and forms a key component of the disinhibition/dysconnectivity hypothesis. Thus, its ability to predict perceptual performance links noninvasive evidence of the dysconnectivity hypothesis to behavior in vivo, for the first time.

### Study Limitations

We recruited a heterogeneous SZ sample, which would be representative and unbiased; however, future studies may wish to recruit separate early and established cohorts for comparison, particularly in light of reports that NMDA receptor hypofunction, and therefore the dysconnectivity hypothesis in general, may be better aligned with early stage than established SZ.^36^

The age range (22–58) could be seen as introducing unnecessary variance to the neuroimaging signals (eg, ref.^37^). However, a broad age range means our sample is heterogenous and inclusive of the individual differences found within the normal population. In this respect, our results should have increased generalizability to the population.

The relative simplicity of the CMC model used in this study was guided by existing literature.^22,38,39^ While complex enough to enable identification of the key synaptic determinants of oscillatory features, it lacks the complexity of multicompartment and detailed spatial models such as the Human Neocortical Neurosolver (https://hnn.brown.edu/).

### Conclusion

In conclusion, these results establish—for the first time—a 4-way construct validity between (1) a visual psychophysical test dependent on local gain control, (2) a noninvasive synaptic assay (DCM), (3) a neurotransmitter deficit (GABA MRS), and (4) the functional anatomy of SZ (in terms of dysconnectivity that speaks to cortical gain control and fast synchronous activity). Crucially, all of these measures offer noninvasive endophenotypes or biomarkers—that may find a powerful application in schizophrenia research.

## Funding

This work was supported by CUBRIC and the Schools of Psychology and Medicine at Cardiff University, the MRC/EPSRC funded UK MEG Partnership Grant (MR/K005464/1). A.D.S. and G.M.W. are supported by a Wellcome Trust Strategic Award (104943/Z/14/Z). K.J.F. is funded by a Wellcome Trust Principal Research Fellowship (088130/Z/09/Z).

## Supplementary Material

sbz066_suppl_Supplementary_Material
