# Supplementary material for: Oscillatory, Computational, and Behavioral Evidence for Impaired GABAergic Inhibition in Schizophrenia
Source: Schizophr Bull. 2019 Jun 20;46(2):345–53. doi: 10.1093/schbul/sbz066 (PMC7442335; doi:10.1093/schbul/sbz066)
Supplement: sbz066_suppl_Supplementary_Material [file sbz066_suppl_supplementary_material.docx]

**Supplementary Materials**

### Inclusion and exclusion criteria

Inclusion criteria for the patient group consisted of a DSM-IV diagnosis of schizophrenia (procedure described in supplementary materials section ‘Participant Assessments‘), being aged between 16-75 years, having English as a first language and having normal or corrected vision. Inclusion criteria for healthy controls included being between 16-75 years of age, having English as a first language and normal or corrected vision. We ensured that all participants had sufficient capacity to give their informed consent to participate. All participants were screened for current drug or alcohol abuse using the MINI interview and excluded if they reported any harmful use of alcohol or drugs. All participants were asked to refrain from any alcohol or drug use 48 hours prior to testing.

Exclusion criteria for all participants were a diagnosis of epilepsy, any severe neurological event such as head injury or stroke and any metal present in their bodies. Additionally, healthy controls were excluded if they or if a first-degree relative had any previous mental health diagnosis of an affective or psychotic disorder (based on the MINI interview for the control participants).

Participant Assessments

Patient participants were recruited via the Cognition in Psychosis study from secondary mental health services on the basis of having a clinical diagnosis of schizophrenia and then underwent a detailed interview including the Schedule for Clinical Assessment in Neuropsychiatry (SCAN ^22^). This information was combined with clinical notes (and when available informant interviews) and experienced raters made OPCRIT ratings and arrived at a consensus diagnosis based on all available information. Inter-rater reliabilities were regularly measured as reported in Lynham et al 2018 (REF). At recall for this study the MINI International Neuropsychiatric Interview ^21^ was administered to confirm a diagnosis of schizophrenia. This information then informed the ratings on the Scale of the Assessment of Positive Symptoms (SAPS ^23^) and the Scale of the Assessment of Negative Symptoms (SANS ^24^). Details of antipsychotic medication were recorded including daily dosages which were standardised across subjects using Olanzapine equivalents as calculated by Gardner and colleagues ^25^.

Control participants were administered the MINI Interview to gather information on any current or past experiences of neuropsychiatric symptoms and disorders, following which control participants were excluded if they had a lifetime diagnosis of any affective or psychotic disorder.

GABA acquisition and analysis

MR data were acquired using a 3 Tesla General Electric Signa HDx scanner with an eight-channel receive-only head RF coil (Medical Devices). Foam padding was used to minimise head movement during scanning. A 5-minute 3D T1-weighted structural scan was obtained for each participant for voxel placement during analysis. One 3x3x3cm³ voxel located in the occipital lobe was scanned using Edited MRS (MEGA-PRESS) for GABA-edited spectra (TE = 68, TR=1800 with a 20ms Gaussian editing pulse applied at 1.9ppm in alternate ON/OFF scans) and a further, unsuppressed water scan was collected for referencing. The occipital voxel was placed medially in the occipital lobe, positioned as posterior as possible but preventing inclusion of the sagittal sinus. See Figure 1A for voxel placement. Acquisition time was ten minutes thirty-four seconds. Quantification of GABA was calculated using Gannet 1.0 toolkit ^1^. Spectra were excluded if the fit error was above 10% with those remaining being corrected for gray matter (GM), white matter (WM) and cerebrospinal fluid (CSF) tissue proportions in relation to water.

MEG paradigm and set up: further details

For the MEG visual grating paradigm, pseudo-random changes in speed are ignored during analysis (as per Hoogenboom ^2^), because any speed-related effects average out across trials. Each experimental run comprised 80 of these trials, and each participant was recorded for three experimental runs (*Radial 1, Radial 2, Radial 3*). See Figure 1A in main text. The stimuli were controlled by Presentation software (Neurobehavioural Systems) and presented using a projector located outside of a magnetically shielded room, which was then projected back onto a screen within the room approximately 60cm from the participants’ eyes at a nominal refresh rate of 60Hz.

Visual orientation discrimination task: further details

Each trial consisted of a fixation dot presented for 250ms, followed by a circular grating test patch, a second fixation spot for 500ms, a second test patch and finally a response fixation spot. The final fixation spot had no time limit and the task only progressed once the participant had responded. The task gave feedback to the participant by beeping once and flashing a green dot should the participant respond correctly to that trial. The difference in orientation between the two test patches in each trial was determined using two randomly-interleaved 1-up 2-down adaptive staircases. Thresholds were calculated from the average of the last 10 reversals of both staircases in log steps, which were then converted to linear units in degrees. Performance measures were calculated as percentage of correct trials (e.g. right / wrong: clockwise or anticlockwise) at each angle of rotation, with chance = 50%.

Neurophysiologically-informed modelling

Dynamic causal modelling for steady-state responses provides a framework for explaining the spectral densities of neurophysiological signals using parameterised computational models. These models use differential equations to model interacting cells, using equations derived from invasive and in-vivo recordings. The model used in this study employed the architecture and convolution equations of Douglas and Martin (1994), which includes 4 interacting, layer-resolved cortical populations. Using DCM to optimise the model parameters, so that the output of the *in-silico* model best matches the real (MEG virtual sensor) spectral density, we can assess synaptic coupling between populations in the model – as well as post-hoc exploring which model parameters determine which spectral features.

Statistical Analyses

For the MRS GABA measures, we used an independent samples t-test to compare the mean GABA concentration in the schizophrenia group with that of the control group.

For MEG spectral features (peak frequency and amplitude), we used a repeated-measures analysis-of-variance (ANOVA) with a group-by-features (control/SZ by frequency/ amplitude) design for transient (0 - 300 ms) and sustained (300 – 800 ms) responses. ANOVAs were run separately for frequency and amplitude.

For the orientation discrimination task, we used a 2x2 ANOVA (group by condition) to compare the mean orientation thresholds of the schizophrenia group with those of the control group, for the oblique and vertical conditions.

For the computational model parameter analysis, we used an ANOVA with a group-by-parameter (8 coupling parameters) design and Bonferroni corrected p-values. We further tested for a group-by-session-by-parameter interaction and parameter-by-session effect in order to rule out an effect of session on between group differences in parameters. To further assess the effects of parameters between groups, we used JASP (JASP Team 2018, Ver 9) to compute parameter Bayes factors.

To assess the relationship of coupling parameters with (1) orientation discrimination thresholds and (2) symptom severity scores (schizophrenia group only), we used Pearson correlations and Bonferroni corrected p-values. Note, for the correlations with orientation thresholds, which were done 3 times, correlations were computed *per-run* to demonstrate repeatability of effects. Parameter Bayes factors were computed for each run and condition.

Note that, while it may have decreased signal-to-noise, computing the correlations between synaptic parameters and performance separately across the three runs of performance measures (rather than by pooling re-runs) allowed us to demonstrate repeatability of the effects, since the correlations were consistent across the 3 runs.

Results: *Occipital GABA levels are reduced in schizophrenia.*

Of the total sample, 26 controls and 24 SZ subjects completed MRS scans for GABA. Of these data, 1 subject from each group was excluded due to GABA measures greater than 3 SD from the group (and total sample) mean. An independent sample t-test revealed a significant reduction in occipital GABA concentration for the SZ group (Controls mean = 1.81 (SD = 0.2), SZ mean = 1.67 (SD = 0.23), p=0.031, Figure 1C). These results were not driven by differences in tissue ratios between groups as confirmed by comparisons of CSF, grey matter and white matter (independent samples t-test; t(49), 0.462, p=0.646, t(49), 0.097, p=0.923 and t(49), -0.160, p=0.874, respectively).

[Supplementary Figure 1]

*Results: No difference in pre-stimulus gamma between groups*

The pre-stimulus spectra in the gamma range (> 30 Hz) was compared between groups across the 3 sessions in order to confirm that the stimulus induced changes in gamma observed were indeed stimulus related, rather than reflecting a baseline shift in gamma power in SZ. Supplementary figure 2 demonstrates that, within the visual gamma range of interest (30 – 70 Hz) there were no differences in pre-stimulus gamma between groups. In session 1, SZ had slightly increased gamma power above 75 Hz (t = 2.6, p = 0.014) while in session 3, SZ demonstrated slightly increased gamma power at 90 Hz (t = 2.2, p = 0.034); however, these (uncorrected) differences are above the frequency range of interest.

[Supplementary Figure 2]

Discussion on GABA

*GABA deficit*

The reduction in GABA levels in the occipital cortex in the SZ group replicates previous reports showing reductions in MRS GABA in this region ^3–5^, tentatively suggesting reduced GABA levels are a repeatable state biomarker of SZ. These reductions may reflect downstream consequences of a reported reduction in the mRNA of the GABA synthesising GAD67 enzyme in SZ ^6^. However, a major caveat to this interpretation is that a recent meta-analysis by Egerton and colleagues ^7^ found no consistent evidence for MRS GABA reductions in SZ across prefrontal, parietal/ occipital cortex and striatum.

In accordance with previous studies that did demonstrate reduced GABA; this study recruited only chronic schizophrenics (~10 years post diagnosis). Consequently, these results could reflect the physiology of predominantly chronic illness, or even the physiological consequences of complex treatment histories. Further work in first-episode, unmedicated, SZ would help elucidate any disease-stage dependent changes in GABA, particularly in light of recent suggestions that NMDA receptor hypofunction may be a characteristic predominantly of early-stage SZ ^8^.

Despite much evidence to support a link between GABAergic inhibition and gamma oscillations ^9–11^, we did not find a correlation between occipital MRS GABA and gamma frequency or amplitude. This is not surprising in light of ﻿Cousijn and colleagues ^12^, who also found no evidence for a correlation between MRS GABA and gamma features in a large sample of 50 healthy individuals. An explanation for this discrepancy may be that MRS GABA levels, which are measures of bulk concentration, are not closely related to GABAergic function. A better marker, then, would be a measure of GABA-A receptor density, which has been shown to correlate with both frequency (positively) and amplitude (negatively) ^10^. Future studies may therefore wish to investigate the relationship of gamma features with PET-derived measures of GABA-A receptor density in individuals with schizophrenia.

*Modelling discussion: predictive coding accounts*

Our modelling results are entirely consistent with theoretical perspectives on predictive coding in SZ. Specifically, it is proposed that many features of SZ, including hallucinations, delusions and sensory attenuation, may be explained by aberrant encoding of precision ^13^. Using the proposed mapping of Bastos and colleagues ^14^, encoding of the precision of prediction errors corresponds to the excitability – or postsynaptic gain – of superficial pyramidal cells under inhibitory control; hence the synaptic parameter identified in our model as able to predict perceptual performance (G11 figure 6a) may represent a physiological corollary of precision encoding. In more general terms, a failure to encode the precision or confidence afforded to ascending prediction errors in predictive coding is a key candidate for explaining the false inference associated with schizophrenia (and several other neuropsychiatric syndromes). In the current setting, this false inference provides a formal account of both positive and negative phenomena; such as a resistance to illusions, and predisposition to hallucinosis and hallucinations.

Supplementary Materials References

1. Edden RAE, Puts NAJ, Harris AD, Barker PB, Evans CJ. Gannet: A batch-processing tool for the quantitative analysis of gamma-aminobutyric acid-edited MR spectroscopy spectra. *J Magn Reson Imaging*. 2013:n/a-n/a. http://doi.wiley.com/10.1002/jmri.24478.

2. Hoogenboom N, Schoffelen JM, Oostenveld R, Parkes LM, Fries P. Localizing human visual gamma-band activity in frequency, time and space. *Neuroimage*. 2006;29(3):764-773. doi:10.1016/j.neuroimage.2005.08.043.

3. Yoon JH, Maddock RJ, Rokem A, et al. ﻿Gamma-Aminobutyric Acid Concentration is Reduced in Visual Cortex in Schizophrenia and Correlates with Orientation-Specific Surround Suppression. NIH Public Access. 2010;30(10):3777-3781. doi:10.1523/JNEUROSCI.6158-09.2010.

4. Kelemen O, Kiss I, Benedek G, Kéri S. Perceptual and cognitive effects of antipsychotics in first-episode schizophrenia: The potential impact of GABA concentration in the visual cortex. *Prog Neuro-Psychopharmacology Biol Psychiatry*. 2013;47:13-19. doi:10.1016/j.pnpbp.2013.07.024.

5. Thakkar KN, Rösler L, Wijnen JP, et al. 7T Proton Magnetic Resonance Spectroscopy of Gamma-Aminobutyric Acid, Glutamate, and Glutamine Reveals Altered Concentrations in Patients With Schizophrenia and Healthy Siblings. *Biol Psychiatry*. 2017;81(6):525-535. doi:10.1016/j.biopsych.2016.04.007.

6. Schahram Akbarian, MD, PhD; James J. Kim; Steven G. Potkin, MD; Jennifer O. Hagman, MD; Alireza Tafazzoli; William E. Bunney Jr, MD; Edward G. Jones, MD P. Gene expression for glutamic acid decarboxylase is increased in prefrontal cortex of depressed patients. *Arch Gen Psychiatry*. 1995;52(4):258-266.

7. Egerton A, Modinos G, Ferrera D, McGuire P. Neuroimaging studies of GABA in schizophrenia: A systematic review with meta-analysis. *Transl Psychiatry*. 2017;7(6):e1147-10. doi:10.1038/tp.2017.124.

8. Anticevic A, Corlett PR, Cole MW, et al. N-methyl-D-aspartate receptor antagonist effects on prefrontal cortical connectivity better model early than chronic schizophrenia. *Biol Psychiatry*. 2015;77(6):569-580. doi:10.1016/j.biopsych.2014.07.022.

9. Wang XJ, Buzsáki G. Gamma oscillation by synaptic inhibition in a hippocampal interneuronal network model. *J Neurosci*. 1996;16(20):6402-6413. doi:citeulike-article-id:134404.

10. Kujala J, Jung J, Bouvard S, et al. Gamma oscillations in V1 are correlated with GABAA receptor density: A multi-modal MEG and Flumazenil-PET study. *Sci Rep*. 2015;5(June):1-12. doi:10.1038/srep16347.

11. Gonzalez-Burgos G, Lewis D a. NMDA receptor hypofunction, parvalbumin-positive neurons, and cortical gamma oscillations in schizophrenia. *Schizophr Bull*. 2012;38(5):950-957. doi:10.1093/schbul/sbs010.

12. Cousijn H, Haegens S, Wallis G, et al. Resting GABA and glutamate concentrations do not predict visual gamma frequency or amplitude. *Proc …*. 2014. http://www.pnas.org/content/111/25/9301.short.

13. Adams RA, Stephan KE, Brown HR, Frith CD, Friston KJ. The Computational Anatomy of Psychosis. *Front Psychiatry*. 2013;4. http://www.frontiersin.org/Schizophrenia/10.3389/fpsyt.2013.00047/abstract.

14. Bastos AM, Usrey WM, Adams R a., Mangun GR, Fries P, Friston KJ. Canonical Microcircuits for Predictive Coding. *Neuron*. 2012;76(4):695-711. doi:10.1016/j.neuron.2012.10.038.
